# Predictors of psychological stress and behavioural diversity among captive red panda in Indian zoos and their implications for global captive management

**DOI:** 10.1038/s41598-022-17872-y

**Published:** 2022-08-18

**Authors:** Aamer Sohel Khan, Stephen E. G. Lea, Piar Chand, Upashna Rai, Nagarajan Baskaran

**Affiliations:** 1 Mammalian Biology Lab, Post Graduate Department of Zoology and Wildlife Biology, A.V.C. College (Autonomous), Mannampandal, Mayiladuthurai, Tamil Nadu 609305 India; 2 Department of Psychology, Washington Singer Laboratories, University of Exeter, Exeter, UK; 3 Padmaja Naidu Himalayan Zoological Park, Darjeeling, West Bengal 734101 India

**Keywords:** Ecology, Zoology

## Abstract

At high elevations, biodiversity is at elevated risk from extinctions due to rapid environmental changes. In the most of its range in Himalayas, the red panda, an endangered species, is struggling to survive in the wild, and a global captive breeding programme has been launched to conserve the species. Because captivity can have negative impacts on animals, reducing the chance of successful reintroduction, we investigated the predictors of stereotyped behaviour and behavioural diversity of red panda (*n* = 26), and the effect of stereotypy on their behavioural diversity in three Indian zoos. Multivariate analysis showed that stereotypy increased with density of logs on the ground, age and higher among pandas in zoo 3 compared to zoo 2, but decreased with number of nests, sociality, tree density and tree height used. Similarly, behavioural diversity increased with log density, but decreased among pandas in zoo 2 compared to zoo 1, during summer compared to winter, and also with ambient temperature, stereotypy, tree density, and tree height used. The relationship between stereotypy and behavioural diversity was negative, but not significant. Provision of a greater density of trees was associated with higher levels of daytime inactivity. Findings from this study have global relevance, as the captive red panda experiences similar welfare issues around the world, and our data provide empirical support for some existing guidelines for red panda husbandry.

Biodiversity is vulnerable to the current rapidly changing environmental conditions. Landscape change and habitat loss cause range shift, leading to a higher extinction rate^1–3^, especially among range-restricted species such as those endemic to high-elevation^4^. Conservation breeding programmes have been criticised^5^, but in some circumstances they offer the only current way of saving rare species, allowing captive stock to be bred for possible reintroduction^4,6^; for a review of the issues see^7^. The ultimate aim of captive breeding is to conserve genetic diversity and re-establish self-sustaining populations in the wild^5^. However, captivity can drastically modify animal behaviour^8^. Furthermore, good animal welfare is essential if the conservation goals of captive breeding are not to be compromised^4^. Captive animals rely on humans for food, water and shelter, and failure to provide such resources reliably will lead to distress^8–10^. Moreover, captive animals cannot control potential stressors such as visitors, temperature, sound, and light levels, which in the wild they could manage or escape^10–13^.

Assessing animal welfare in captivity is often difficult^14^, and behaviour is an important tool for doing so, because behavioural change is the first line of defence against changing environmental conditions^15^. A common method for assessing an animal’s welfare is to examine how the behaviour it exhibits varies with housing and management conditions^16,17^. A particular concern is unusual behaviour among captive animals, which has not been observed in their wild conspecifics^7^, especially stereotyped behaviour. Stereotypy typically consists of repetitive or invariant movements with no obvious goal or function^18^. At least initially, these appear to be due to frustration^19^, and constitute a coping mechanism against environmental stressors^19,20^. Examples include pacing, over-grooming, licking inedible objects, and head bobbing^7^. Stereotypy is associated with inappropriate environmental conditions, lack of stimulation, small enclosure size and lack of environmental enrichment. These variables will alter animals’ activity and behaviour in captivity^10^, but once established, stereotypy may persist despite improvements in housing conditions^21^. Although not all repetitive behaviours reflect some sort of distress, stereotypic behaviour is always related to issues of animal welfare^22^, and was indeed one of the first behavioural cues to be used as an indicator of poor welfare in captivity^14,16^.

Another relevant indicator of welfare in captive animals is behavioural diversity^17,18^, because maintaining a wide range of natural behaviour is essential if reintroduction is to stand a chance of success. Although higher behavioural diversity is typically taken as a positive welfare indicator, this may not be appropriate for all species^19^. For a crepuscular, nocturnal or mostly inactive species, lower behavioural diversity during day time might be a sign of good welfare. Diversity and stereotypy may not be independent: the relation of behaviours like choice of food, feeding, and sociality to stereotypy has been considered in the past^23–25^, but the relationship between stereotypy and behavioural diversity is not clear, most often, increased stereotypy is associated with lowered behavioural diversity, but there are many exceptions^19^.

The present paper focuses on the red panda (*Ailurus fulgens*). There are two living subspecies (or species^26^), *Ailurus fulgens fulgens* and *Ailurus fulgens styani*^27^. In the wild, red pandas fit the description of a largely inactive, crepuscular or nocturnal species^20,28^, although in captivity they have shown diurnal activity patterns^29^, so the relation between stereotypy, diversity and welfare in captive red pandas offers an interesting topic for investigation. They are small omnivorous mammals with several adaptations to feeding on bamboo, and inhabit the whole of the Eastern Himalayan ranges in Nepal, Bhutan and India, extending into eastern China and Myanmar, normally at elevations of 1500–4000 m^22^. The species is endangered, with a wild population that may be below 2500 mature individuals and on a declining trend^27^, and insufficient protected areas^30^. Globally, ex situ conservation aims to support the species through captive breeding programme to make reintroduction possible^31^. Indian breeding centres, at the time of study, housed 26 individuals. Although substantial guidelines for the care of red pandas in captivity have been developed, they are based on accumulated experience rather than controlled observation^32,33^. A recent review stresses the need for more research not only in free ranging red panda populations, but also in the range country captive population^34^. Red pandas are annual breeders with a low reproductive rate in the wild^31^. The cause of their reproductive decline, as also their breeding biology and what stressors may be acting on it, are still unknown. Stress inhibits reproduction through a variety of mechanisms, which appear to be species-specific^35^. The need is to establish the species’ behaviour patterns and any behavioural indications of poor welfare in captivity^36^.

Little has been published on the behaviour of red pandas in captivity. One study used pandas kept in 13 zoos in the United Kingdom and Ireland, recording the levels of stereotypical behaviour in each zoo^37^. Another assessed personality in pandas in New York zoos^38^. In India the Darjeeling zoo has reported data on the activity and feeding pattern of captive pandas in summer^29^, the only study on Indian captive population so far. This study aims to provide an empirical basis for the current guidelines^39^ and good practices promoted by several zoo authorities for red panda management, as affirmed in a survey study^40^.

The ultimate goal of captive breeding is reintroduction to support wild populations. Individuals intended for reintroduction must be behaviourally competent to survive in the wild. Understanding and assessing the welfare in captive individuals would help breeding programmes produce healthy, competent individuals with naturalistic behaviour, suitable for reintroduction. By evaluating the extent of stereotypy and behavioural diversity among 26 captive pandas managed at three different Indian zoos, we aimed to assess the drivers of psychological stress and behavioural diversity in red pandas, and the relationship between them.

Our findings have implications for management and husbandry practices of captive red pandas and hence the effectiveness of captive breeding programmes for successful reintroduction.

## Results

Among 26 red pandas observed during daylight hours, 24 individuals showed stereotypic behaviours. Pacing, tongue flicking and position circling were the three behaviours observed. Position circling was observed among 14 individuals at Zoo 1 (54% of total assessed population across three zoos); this is the first report of this behaviour in pandas. The mean proportion of time spent in stereotyped behaviour was 2.0% ± 0.625% (54.5 min), with a mean behavioural diversity index of 1.07 ± 0.032 (using Shannon’s *H* measure). Both stereotypy and behavioural diversity varied with some of the biological and environmental factors, and also differed between zoos. In the current study the overall activity budget of pandas was: inactive 59%, non-stereotyped activity 39% and stereotypy 2%.

### Extent of stereotypic behaviour in relation to biological and environmental factors: univariate analysis

**Figure 1:**
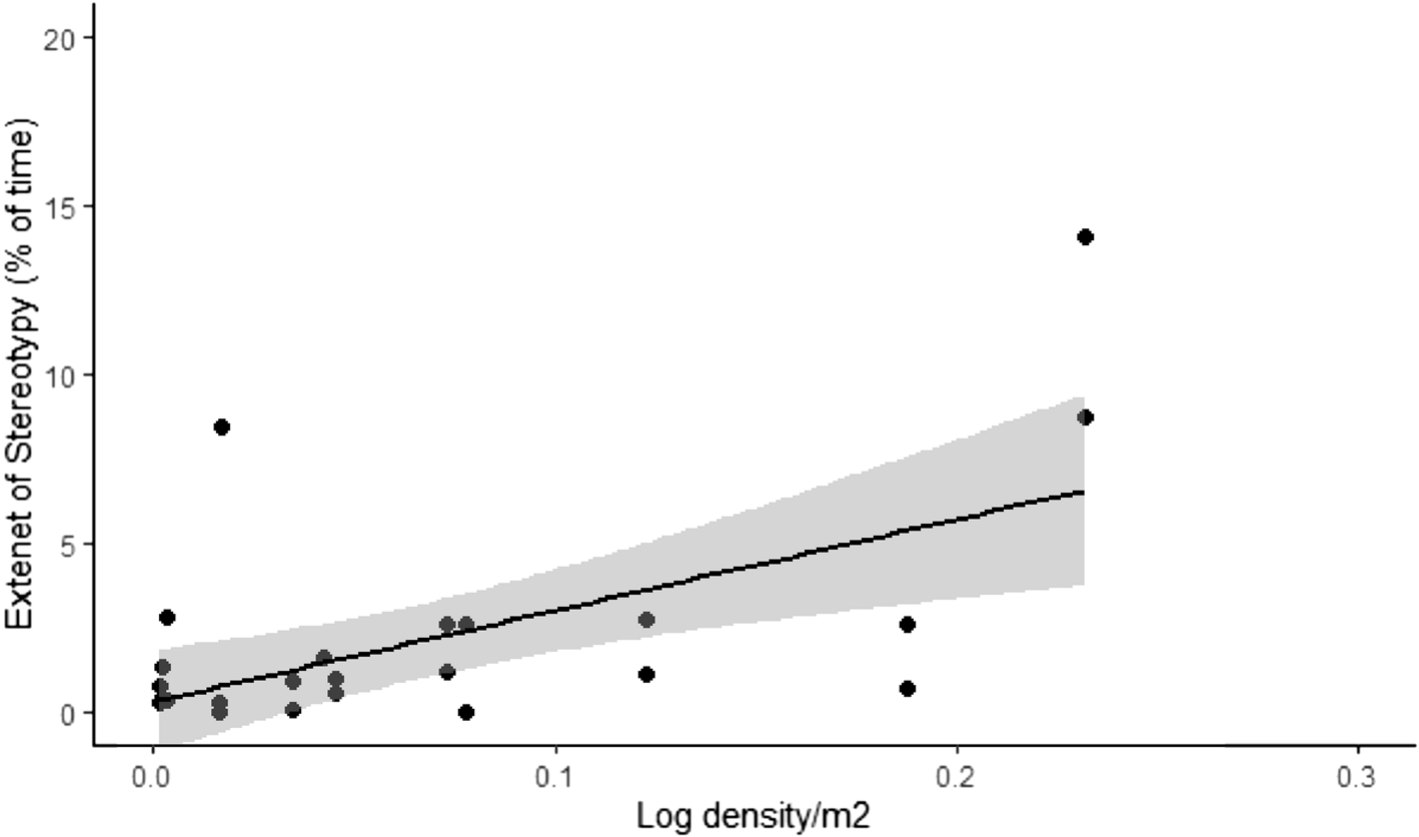
Relation between stereotypy and log density on ground in captive red pandas in Indian zoos. The grey ribbon indicates standard error.

Among the 15 independent factors tested against the extent of stereotypy, age, zoo and frequency of feed showed significant variation on univariate tests (Table 1). For example, pandas showed significant variation in stereotypy across the three different zoos (*p* < 0.05), with the lowest level in Zoo 2 followed by Zoo 1 and the highest in Zoo 3. Further, Scheffé’s post-hoc test revealed that there was no significant difference in stereotypy between Zoo 1 and Zoo 2 (*p* > 0.05), while the individuals in Zoo 3 stereotyped more than that of Zoo 1 (*p* < 0.05) and Zoo 2 (*p* < 0.05). The extent of stereotypy was higher in pandas fed once compared to those fed twice (*p* < 0.05). Sub-adult pandas showed more stereotype than adults and cubs (*p* < 0.05). Stereotypy increased significantly with the density of logs on the ground (*β* = 27, R^2^ = 0.32, F = 12.63, *p* < 0.05, Fig. 1); however, we found that the relation between log density and stereotypy might be deceptive, because four individuals that showed highest stereotypy were housed in small enclosures with no trees but more logs on the ground. Removing those four individuals and running linear regression showed that the relation was not significant (*β* = 1.60, R^2^ = 0.002, F = 0.043, *p* > 0.05).

**Table 1:** Extent of stereotypy and behavioural diversity in relation to biological and environmental factors among captive pandas (n = 26 and n = 24 respectively) in Indian zoos: univariate analysis.

Variables	Categories	Stereotype		Behavioural diversity	
		% of time spent per day	Test, & (*p*) value	Shannon–Wiener *H*	Test, & (*p*) value
		Median ± IQR (n)		Median ± IQR (n)	
**Biological**					
Age	Adult	1.08 ± 1.94 (19)	*χ*^2^ = 4.56	1.04 ± 0.22 (17)	*χ*^2^ = 0.18
	Sub-adult	11.37 ± 2.67 (2)	*df* = 25	1.06 ± 0.07 (2)	*df* = 23
	Cub	0.06 ± 0.00 (5)	(**0.033**)	0.95 ± 0.31 (5)	(0.916)
Sex	Female	0.70 ± 1.81 (15)	*U* = 62	1.02 ± 0.28 (14)	*U* = 62
	Male	1.16 ± 1.99 (11)	(0.287)	1.04 ± 0.15 (10)	(0.639)
Sociality	Paired	0.88 ± 2.27 (25)	*U* = 2	1.02 ± 0.23 (23)	*U* = 10
	Single	8.42 ± 0.00 (01)	(0.161)	1.08 ± 0.00 (01)	(0.828)
**Environmental**					
Season	Winter	0.62 ± 2.51 (18)	*U* = 61	1.03 ± 0.29 (16)	*U* = 48
	Summer	1.47 ± 1.10 (08)	(0.243)	1.03 ± 0.01 (08)	(0.327)
Zoo	Zoo 1	0.88 ± 1.23 (17)	*χ*^2^ = 9.42	1.08 ± 0.27 (17)	*χ*^2^ = 6.32
	Zoo 2	0.08 ± 0.12 (03)	*df* = 25	0.85 ± 0.11 (03)	*df* = 23
	Zoo 3	2.66 ± 5.73 (06)	(**0.009**)	0.92 ± 0.18 (04)	(**0.043**)
Feeding frequency	Once	2.66 ± 5.73 (6)	*U* = 21	0.95 ± 0.21 (4)	*U* = 26
	Twice	0.66 ± 1.18 (20)	(**0.017**)	1.06 ± 0.21 (20)	(0.278)
Quantum of bamboo	High	0.88 ± 1.23 (14)	*U* = 77	1.08 ± 0.19 (14)	*U* = 58
	Low	1.08 ± 2.47 (12)	(0.718)	0.95 ± 0.28 (10)	(0.482)

For more detailed definitions of variables, see Table 5.

*U* and *χ*^2^ indicate the test statistics from the Mann–Whitney U and Kruskal–Wallis H tests respectively; df is degrees of freedom and IQR is interquartile range. Variables showing significant effects are highlighted.

Significant values are in bold.

### Behavioural diversity in relation to biological and environmental factors: univariate analysis

**Figure 2:**
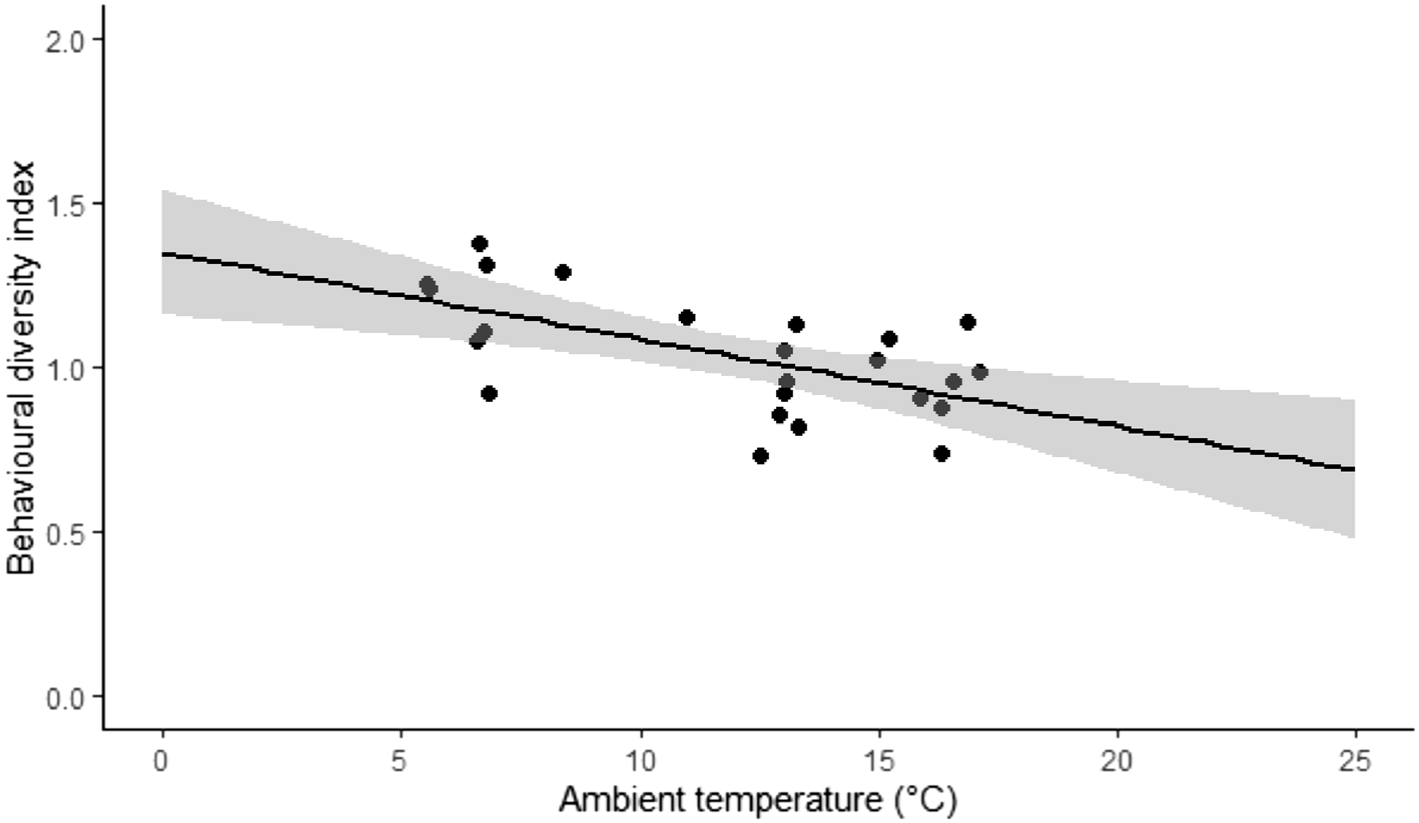
Relation between behavioural diversity index and ambient temperature in captive red pandas in Indian zoos. The grey ribbon indicates standard error.

**Figure 3:**
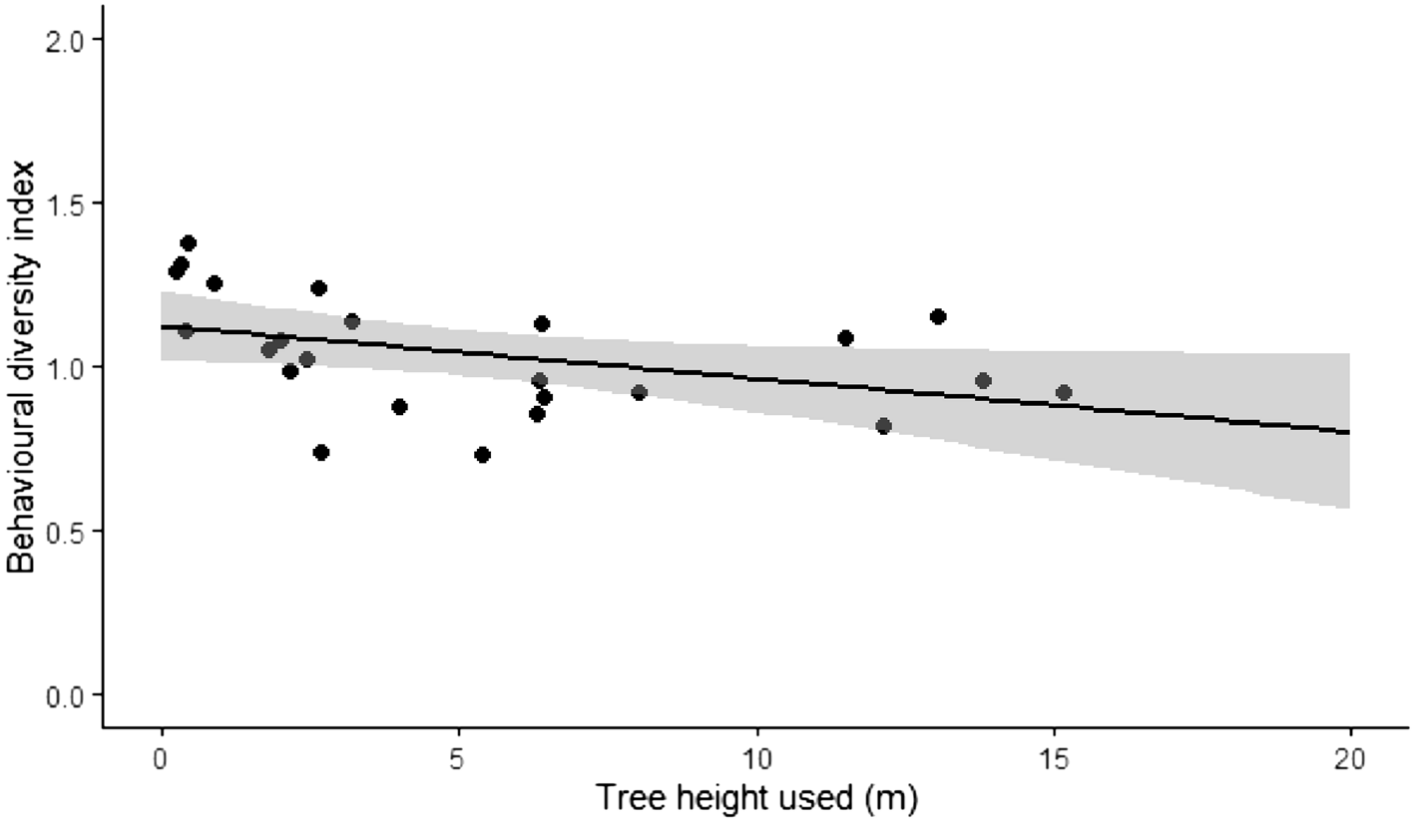
Relation between behavioural diversity index and tree height used by pandas in Indian zoos. The grey ribbon indicates standard error.

Among the independent factors tested, behavioural diversity varied significantly only with zoo (Table 1). The pandas managed in different zoos exhibited different levels of behavioural diversity (*p* < 0.05). Analysis using Scheffé’s test showed that behavioural diversity differed significantly only between the individuals in Zoo 1 and Zoo 2 (*p* < 0.05). Univariate linear regression revealed that behavioural diversity decreased with ambient temperature (*β* = − 0.026, R^2^ = 0.37, F = 13, *p* < 0.05, Fig. 2), enclosure area (*β* = − 7.78e−5, R^2^ = 0.24, F = 7, *p* < 0.05) and tree height used by pandas (*β* = − 0.16, R^2^ = 0.18, F = 4.80, *p* < 0.05, Fig. 3).

### Influence of biological and environmental factors on stereotypy and behavioural diversity: multivariate analysis

REVS analysis yielded a model with seven predictors significantly related to the extent of stereotyped behaviour (Table 2). Stereotypy showed increasing trends with density of logs on the ground, age and higher among pandas in zoo 3 compared to that of zoo 2, but showed declining trend with number of nests, sociality, tree density and tree height used by pandas (*p* < 0.05). Together, these seven predictors explained 86% of the variations in the extent of stereotypy among captive pandas in Indian zoos.

**Table 2:** REVS model to explore the effect of biological and environmental factors on the intensity of stereotype and level of behavioural diversity among captive pandas in India zoos.

Dependent variable	Factors	Estimate ± SE	*t*	*p*	AIC (∆AIC)	Model *p*	Adj. R^2^
Stereotype	(Intercept)	16.78 ± 2.522	6.65	**<** **0.001**	17.26		0.86
	Log density	59.59 ± 6.920	8.61	**<** **0.001**	(0.000)	**<** **0.001**	
	Number of nests	− 1.47 ± 0.393	− 3.75	**0.001**			
	Sociality	− 7.64 ± 1.397	− 5.47	**<** **0.001**			
	Zoo 3 compared to Zoo 2*	1.23 ± 0.501	2.46	**0.023**			
	Tree density	− 16.38 ± 5.957	− 2.75	**0.013**			
	Age	0.18 ± 0.079	2.29	**0.034**			
	Tree height used	− 0.24 ± 0.071	− 3.43	**0.002**			
Behavioural diversity	(Intercept)	1.55 ± 0.178	8.72	1.55e−6	− 113		0.79
	Log density	4.44 ± 0.827	5.36	**<** **0.001**	(0.000)	**<** **0.001**	
	Zoo 2 compared to Zoo 1**	− 0.90 ± 0.208	− 4.31	**0.001**			
	Season: summer compared to winter	− 0.65 ± 0.194	− 3.35	**0.005**			
	Ambient temperature	− 0.082 ± 0.0260	− 3.14	**0.008**			
	Stereotypy	− 0.023 ± 0.0102	− 2.77	**0.017**			
	Tree density	− 14.32 ± 4.459	− 3.21	**0.007**			
	Distance to cage mate	0.014 ± 0.0100	1.45	0.170			
	Visitors	0.0006 ± 0.0009	0.77	0.455			
	Tree height used	− 0.014 ± 0.0065	− 2.24	**0.044**			
	Age	− 0.017 ± 0.0086	− 2.00	0.069			
	Quantum of bamboo	0.08 ± 0.0643	1.25	0.232			

Significant values are in bold.

*Zoo 1 kept as reference category.

**Zoo 3 kept as reference category.

For behavioural diversity, the REVS analysis yielded a model with eleven predictor variables (also shown in Table 2). Behavioural diversity was lower among individuals in zoo 2 compared to that of zoo 1, during summer than in winter, and decreased with ambient temperature, stereotypy, tree density and tree height used by pandas (*p* < 0.05). The remaining four predictors in the model were not statistically significant. These eleven factors together explained 79% of the variations in behavioural diversity.

## Discussion

### Influence of independent variables on the extent of stereotyped behaviour

The overall level of stereotypy we observed was low, suggesting that the pandas in our study were not seriously stressed. The variables that we found to be correlated with stereotypy are consistent with what we know of pandas’ natural history. Our study reports that variables like logs on the ground, nest, sociality, zoo, tree density, age and tree height used by pandas are the driving force for stereotypy in captive pandas involved in the study.

Making the captive environment more naturalistic by integrating enrichment into the enclosure seems to be a promising way of alleviating stress and improving both welfare and reintroduction success^41^. It also helps to improve reproductive rate and overall health^39^. Improved health reduces stress and gives greater control over the environment increasing the chances of survival and longevity both in captivity and following release into the wild^5^. It is generally accepted that enrichment of the captive environment increases animals’ ability to cope with challenges and positive use of the environment reduces or eliminates aberrant behaviour^23^. Lack of enclosure enrichments and less complex enclosures can cause stereotypy and other atypical behaviours^24^, while providing enrichment increases the frequency of natural behaviours^25^ and thereby reduces stress, which in turn decreases stereotypy^27^. But enrichment needs to be appropriate for the species of animal concerned. Abnormal behaviours are often associated with captive conditions that deviate greatly from the species’ natural environment. Consistent with this argument we found that though dead and fallen logs on the ground are one of the important characteristics of the panda habitats in the wild^42–45^, merely providing them in captivity does not ensure the species’ welfare: in fact, stereotypy increased with log density in our study subjects. This could be due to the fact that four individuals that showed more stereotypy were housed in the small barren enclosures with no trees but more logs as a part of enrichment. Without those four individuals, the linear relation between stereotypy and log density was not statistically significant. This clearly suggested that merely providing logs in the small enclosures does not maintain welfare.

When animals are housed in enclosures designed to resemble their natural habitat by considering their natural history (provision of vegetation, shelter, pool, etc.), there is a reduction or elimination of abnormal patterns of behaviour such as stereotypies, increased fitness and improved health, all of which may influence reproduction^25,46–48^. For many species, nests, shelter or burrows in enclosures will serve as retreat and hiding places, which are essential to cope with environmental stressors^10^. Gerbils, mice and rabbits have all shown less stereotyped behaviour when retreats are provided^9,49–51^. Such retreats can mitigate the effects of zoo visitors, who can serve as a source of stress for species that rarely interact with humans in the wild. Consistent with these previous results, we found that with provision of nests, the extent of stereotypy decreased in captive pandas. Many species prefer nests both for rearing the young as well as for resting and shelter, and pandas follow this pattern, so providing nests in adequate numbers will supports their natural behaviour as well as provide relief from environmental stressors. Zidar recommends providing one more nest than there are individuals in an enclosure^52^.

Although pandas are an asocial species, our study showed that pandas show more stereotyped behaviour when housed alone than when with another individual or in group. Being a solitary species in the wild might encourage management to house them singly in captivity, but not every activity and habit of species in the wild can be used in captivity. For example, polar bears are also a solitary species, and it was at one time thought best to manage them alone, but it was found that managing them in a social setting reduces stereotypic pacing behaviour^53^, consistent with this study. Importantly, managers of zoo should note that living in group is greatly influenced by the individuals’ compatibility and hence this should be kept in mind while pairing.

Similarly, we found that the presence of trees, and greater mean tree height use by pandas, reduced stereotypy. Pandas’ preferred high elevation habitat is favourable for taller trees^20^, and Shrestha et al. found that canopy cover was an important factor in habitats for pandas in the wild^54^. In European zoos, pandas spend 90% of their time off the ground^37^. Consistent with these previous findings, our study reveals that more and taller trees support natural behaviours in panda. The Central Zoo Authority (CZA) of India enrichment manual recommends taller tree provision in panda enclosures, and again we provide empirical support for its recommendation.

We found that with increasing age stereotypy increased in pandas. The older the individuals the more time spent in captivity with its associated risks of stereotypic behaviour. The same trend has been observed in other species: for example in captive bears stereotypic behaviour increased with age^55^. In another study Asiatic black bear and sun bear showed more stereotypy with age^56^.

### Influence of independent variables on behavioural diversity

As noted in the “Introduction” section, in a species like the panda, high daytime behavioural diversity is not necessarily a positive indication of good welfare. However, our comparison of behavioural diversity with stereotypy showed a negative trend (though not significant), suggesting that low behavioural diversity might be associated with poorer welfare.

Nonetheless, we found some results that suggested that lower diversity might in fact be associated with a more natural lifestyle. Because of the amount of time that wild pandas spend foraging^57^ and sleeping or inactive, they cannot show much behavioural diversity, and in our sample of captive individuals, they showed the same trend. For example, behavioural diversity was lower when pandas were provided with more trees in the enclosure. This suggests that when appropriate conditions are maintained in captivity, panda prefer to be inactive during the day, as is consistent with their natural history^57^. As pandas are essentially arboreal mammals, naturally they also spend most of the time inactive (e.g. sleeping) on the trees^57^. Indeed, providing larger trees would promote inactive behaviours and hence lower behaviour diversity in captivity, this captures their natural behaviour. This is consistent with our results where increased tree height used by pandas decreased behavioural diversity.

We found behavioural diversity was greater when there are more logs in the enclosure. In the Yele Reserve in Sichuan, China, Wei et al. found 107 of 185 panda dropping sites (57.8%) on shrub branches, 49 (26.5%) on fallen logs, and only 29 (15.7%) on the forest floor^44^. Droppings were found mostly on elevated structures ranging from 1 to 3 m above the forest floor and occasionally on trees over 12 m. Moreover, microhabitats selected by pandas were also characterized by fallen logs and tree stumps^42,45^. Wei and Zhang mention that to access bamboo leaves easily, pandas usually use some elevated objects, such as shrub branches, fallen logs, or tree stumps to lift their body^43^. Hence, providing tree logs in the vicinity supports their natural behaviour. But at the same time management should keep in mind that merely providing logs in the enclosure would not guarantee species welfare, as discussed in previous section with respect to stereotypy.

Temperature is an important element of microclimate for animals, and influences the activity level of captive animals^10^. When temperature rises, many species show distress in captivity^10^. The red panda inhabits low-temperature areas^20^, so it is unlikely that higher temperatures would support natural behaviours. We found that with increased temperature behavioural diversity decreased in captive pandas. Similarly, we found that pandas showed higher behavioural diversity in the winter season, where temperatures are low as compared to summer season.

Studies that have tried to relate behavioural diversity and stereotypy in captive animals have varied in their interpretation; many have found significantly inverse relationships between the two^19^. In this study our multivariate model suggested that behavioural diversity is negatively influenced by stereotypy in captive pandas, confirming previous research.

Other factors associated with variations in behavioural diversity are less easy to identify with welfare, positive or negative. Behavioural diversity also decreases with age of pandas and increases with distance to cage mate, number of visitors and quantum of bamboo provided, though these effects were not significant in the REVS model.

Taken together, these results suggest that higher behavioural diversity is not a straightforward indicator of better welfare in all captive animals. The overall non-significant relationship between stereotyped behaviour and diversity we observed could well be the result of a mixture of factors operating in opposite directions. To interpret diversity correctly, it would be helpful to know what level of diversity the species shows in the wild, and such data are rarely available—a limitation of our study as of many others. Although there are dissenting voices^58^, arguably what matters most both in terms of welfare and in terms of potential reintroduction to the wild, is that a captive animal’s time budget approximates as closely as possible that of a wild animal. It is not diversity as such that is important, but the behaviours that the animal exhibits.

#### Differences between zoos

Our study showed that both the extent of stereotyped behaviour and behavioural diversity varied significantly among zoos. However, Zoo 2, an important breeding centre, housed only a female and her two cubs; this may lead to many factors being confounded and is thus a limitation to our study. Captive animals rely on the zoo environment, its routine and husbandry practices to limit their stress levels, and any failure to provide suitable resources will certainly disturb them and lead to distress^10^. Controlling such variables appropriately will help reduce stress among captive animals, and we can rely to some extent on our knowledge of the species’ natural history to guide us through this challenge. Our study was able to identify some of the factors that are associated with better welfare, but even with these factors taken into account, significant differences among the three zoos remained. These are presumably due to subtler variations in the zoos’ environment or management regimes. Since the panda is endemic to high elevations, we considered whether differences between the elevations of the zoos might be relevant, but the biggest differences were between Zoos 1 and 3, which are at essentially the same elevation.

In Zoo 1 pandas showed lower stereotypy and higher behavioural diversity then the other two zoos. Again, these differences may be due to subtle differences between the management regimes in the three zoos; possibilities include keepers’ attitudes and the zoo’s experience in managing pandas. It is notable that Zoo 1 has longer and wider experience in the management of red pandas than the other two zoos, which have joined the captive breeding programme more recently and have fewer animals. Other notable differences were that in Zoo 1, pandas are fed twice a day as compared to the other two zoos where feed is given all at one time (both bamboo and supplementary diet); and that in Zoo 1 the enclosures were of a good size for a small mammal like the red panda, and were well maintained with much natural vegetation. The other two zoos had a large enclosure with poor vegetation (trees and grass), or a small enclosure with a barren floor and no trees at all. Location of the enclosure also needs to be considered: in two of the enclosures at Zoo 3 the sun shone directly on the animals with no shade as such, keeping the temperature higher than would be natural for pandas. Any of these factors could be the reason the pandas performed comparatively well in Zoo 1, and it would be necessary to study a wider (and, therefore, cross-national) sample of zoos holding pandas to identify which of them are the most important.

## Conclusions

We conclude that maintenance of adequate number of nests, more and higher trees and log density in the enclosure will reduce stereotypies among captive pandas to a considerable extent. Similarly, encouraging pandas to spend more of the daytime in feeding and resting by providing proper enrichment will support their natural behaviour. It is notable that these factors are also important habitat attributes for pandas in the wild^54^. Our study is important as the first to provide detailed empirical study of the welfare of captive pandas, and factors influencing it in the Indian subcontinent, where a natural population exists. A limitation of this study is that there are no comparably detailed studies among panda populations in captivity as well as from the wild around the world. For captive breeding purposes, individuals are exchanged among almost every participating breeding centre in the world (Darjeeling Zoo Management Plan), and in the process individuals with welfare problems will also be transferred. Ultimately this can reduce the success of the expensive global breeding programme. The Indian captive breeding programme holds especial potential because its sites are within the species’ natural range. Its primary responsibility is to maintain the species in the wild through reintroduction. At several points our study provides empirical support for the recommendations of the Indian CZA, and as captive red panda populations experience similar welfare issues among breeding centres around the world, the findings from the present study could help to refine the captive protocol to enhance the welfare of pandas at captive breeding centres in India and elsewhere in the world. In brief, we recommend.

- Enough nests (at least one more than the number of animals in the exhibit) should be provided in the enclosure, as a retreat, especially during the breeding season, where rearing young ones is an essential part of the species’ survival.
- To support species-natural behaviour, and replicate wild conditions, panda enclosures should be enriched with an adequate number of tall trees and multiple tree logs or stumps in a novel, yet complex way with a number of different pathways for simultaneous use by housed individuals.
- Feed should be provided at different time intervals at least twice a day.

Although we did not find a significant relation between behavioural diversity and stereotypy, our multivariate analysis has suggested that stereotypy would affect behavioural diversity inversely in captive pandas. We suggest studying in detail the relationship between behavioural diversity and stereotypy with respect to captive pandas.

## Method

### Study sites

The study was carried out at three different Indian zoos that are part of captive breeding programme (Table 3). More details about the zoo, subjects and their husbandry practices are given in Supplementary material 1.

**Table 3:** Details of various study sites and study individuals observed in the present study.

Study sites (geo-coordinate)	Elevation (m)	Temperature range (°C)	Area in hectares	Study period	No. of Individual			Age cub/sub-adult/adult	No. of enclosure
					♂	♀	Total		
Padmaja Naidu Himalayan Zoological Park (27.05° N 88.25° E)	2134	0–25	27.5	May–July 2017; Dec–Feb 2018	7	10	17	3/0/14	9
Sikkim Himalayan Zoological Park (27.34° N 88.62° E)	1780	4–22	230	March 2018	1	2	3	2/0/1	1
Pt. G. B. Pant High Altitude Zoo (29.38° N 79.46° E)	2100	3–25	5	April 2018	3	3	6	0/2/4	3
Total					11	15	26		13

### Ethics statement

Following Indian national guidelines on working with zoo animals, we first obtained permits from zoo directors, who after evaluating the non-invasive methods of direct observation granted permission. The study observed the subjects during their routine husbandry practices. Research permit number 410/TECH/S.O.65/ZOO-VOL/PNHZP/17–18. *Subjects and data collection* The study involved behavioural sampling of all 26 captive pandas (Supplementary material) that were under the global conservation breeding programme, using focal sampling^59^. All individuals were observed between May 2017 and April 2018. Observations were restricted to the period from 06:00 to 18:00, with each hour divided into four 15-min time slots. Each slot contained a10-min observation period followed by a 5-min gap. During observation, we recorded the time spent on each behaviour to the nearest second. We planned to observe each subject for 6 h on each of four separate days, twice between 06:00 and 12:00 and twice between 12:00 and 18:00; for two individuals only 20 h observations could be collected because of bad weather. In total, 972 h of observations were made. All observations were made by the first author.

#### Evaluation of extent of stereotypy and behavioural diversity

Before initiating the study, we sketched a detailed behavioural ethogram for pandas using a few individuals at Darjeeling zoo via focal sampling^59^ (see Table 4). We also took into account the zoo literature on red pandas, and discussions with zookeepers at the study sites, while preparing the ethogram. To confirm the credibility of the ethogram we compared it with international literature on panda behaviour^37^. We divided the behaviour of the panda into active, inactive and stereotyped behaviours, using the ethogram (Table 4). During observation, when the subject went out of the observer’s sight, we recorded it as 'out of sight' (mean 0.64%, range 0–5%). We calculated the percentage of time spent on stereotyped behaviour for individual animals to compare with the independent factors.

**Table 4:** Ethogram of captive panda at three zoos in India.

Behaviour	Description
**Active**	
Feeding	Feeding on bamboo leaves provided or supplementary diet
Moving	Moving on the trees
Climbing	Climbing up and down the trees and onto logs on the ground
Walking	Walking on the ground with four legs
Grooming	Cleaning their coat or body by licking
Scratching	Scratching body by using paws
Playing	Energetic activities with self, family member or engaging objects like tree logs or trunk, with no obvious immediate goal
Watching	Watching with no obvious concern
Vigilance	Continuous sensory tracking of the environment or events
Defecating	Defecating or urinating
Exploratory	Exploratory/territorial investigation of enclosure, can involve sniffing, digging, interaction with furnishings within the enclosure
Aggression	Threatening or harmful behaviour towards conspecific or keeper
Yawning	Hand stretches at right angles to body and mouth wide open
Stretching	Body stretches with support of tree trunk or log
Cleaning	Vigorous shaky movement of the body
Smelling	Smelling around the tree log or enclosure area
Scent marking	Smelling and rubbing anal gland either left to right or up and down at particular site in the enclosure or on trees
Roll down	Rolling the body apparently playfully
Vocalisation	Vocal call to conspecific or grunt call during aggression
Sniffing	Sniffing around
**Stereotypy**	
Pacing	A repetitive locomotion between two fixed points
Tongue flicking	Moving tongue in and out multiple times while resting or sitting idle
Position circling	Standing and changing position by circling at the same place, making a complete circle or semi-circle
**Inactive**	
Sleeping	Sleeping gesture either curling the body or hand spread out
Resting	Laying down at trees with no activity
Sitting	Sitting idle

#### Behavioural diversity calculation

We measured behavioural diversity for each individual using the Shannon–Wiener diversity index H^18^ using the formula:

$$H = - \sum_{i} \left( {p_{i} \ln p_{i} } \right)$$

where *p*_*i*_ is the proportion of the 24 h (or 20 h) observation time that the individual spent performing the *i*th behaviour. A higher value of *H* (in this study it ranged from 0.45 to 1.37) represents greater behavioural diversity, resulting either from a larger number of behaviours being emitted, or from a more even distribution of time between a given number of behaviours.

#### Assessing biological and environmental factors influencing stereotypy and behavioural diversity

To test the influence of various biological and environmental factors on the extent of stereotypy and behavioural diversity, we assessed the 15 variables listed in Table 5. In the case of behavioural diversity, we included the extent of stereotypy as an additional independent variable. We have included season as one of the factors, owing to significant difference in ambient temperature between winter (as low as zero) and summer (as high as 25 °C), with which the pandas alter their behaviours. Accounting season as one of the variables reflects this behaviour adaptation if any. The season considered in the study represent the actual observation time and its effect on the behaviour.

**Table 5:** List of dependent and independent (environmental and biological) variables.

Name of variable	Description of variable
**Dependent**	
Stereotypy	Proportion of time spent on unusual behaviours or performance of a repetitive activity with no obvious goal or function
Behavioural diversity	Calculated by incorporating the proportion of behavioural diversity in Shannon–Weiner diversity index H (Shannon and Weaver 1949)
**Independent (biological)**	
Sex	Male/female
Age	Cub (0–12 months), sub-adult (12–24 months), adult (> 24 months)
Sociality	Single or pair
**Independent (environmental)**	
Season	Individuals observed in winter were coded as ‘0’ and summer as ‘1’
Number of visitors	Total number of visitors to the enclosure during the observation, visual count
Ambient temperature	Temperature of surrounding in °C during the observation, recorded using temperature sensor
Enclosure area (m^2^)	Estimated two-dimensional area of the enclosure (obtained from zoo records)
Zoo	Zoos are different in terms of their management practices
Tree height (ocular method using measured reference points)	Tree height at which the red panda was spotted for different activities during observation
Distance to cage-mate (ocular estimation by observer at the beginning of each observation)	Distance maintained by the animal from its cage-mate housed in the enclosure
Log density	No. of logs/m^2^ placed on the ground or made into climbing structure within the enclosure as an enrichment for animal activity were counted and arrived at density using area of enclosure
Tree density	Number of mature live trees (≥ 20 cm girth at breast height) in each enclosure was counted and arrived trees/m^2^ using enclosure size
Frequency of feeding	Number of times feed given per day
Quantum of bamboo	Bamboo given per day (low: < 4 kg/individual/day, high: ≥ 4 kg/individual/day) as per the husbandry practices of the zoos
Provision of nests	Number of nests, a closed wooden nest with an entry point, accessible to the animals at all the time

#### Statistical analysis

Statistical analysis was carried out using SPSS for Windows (*Version 16*) and R (*Version 3.4.1*). Before testing for differences in the levels of stereotypy and behavioural diversity due to various biological and environmental variables, we checked the data for normality. The data for the dependent variables (stereotypy and behavioural diversity) were not normally distributed, so we performed non-parametric tests. For the behavioural diversity data set we excluded two outlier data points with respect to the tree density variable. The univariate dependence of stereotypy and behavioural diversity on biological and environmental factors was tested using the Mann–Whitney U test (for factors with two categories) and Kruskal Wallis H test (for factors with three categories). For the season category (winter and summer) we coded subjects as 0 and 1; 0 for winter observation and 1 for summer season. The first-order effects of the continuous variables were checked using simple linear regression; in particular, to check the effect of stereotypy on behavioural diversity, we treated the former as independent variable and latter as dependent variable in linear regression. To quantify and assign empirical support to the simultaneous effects of each of the biological and environmental factors, we employed REVS (Regression with Empirical Variable Selection) analysis^60^, a branch and bound all subsets regression technique (implemented by the LEAPS package in R). REVS analysis can handle collinearity^60^ therefore we did not test our data for collinearity. Because of the limited size of the dataset, it was not feasible to examine interactions between independent variables. The REVS method selects the best model based on R^2^ and delta-AIC values. Where categorical variables had more than two values, we used the value with most sampled individuals as the reference category.

## Supplementary Information

Supplementary Information.

## Data Availability

Full data used for analysis, the R script used to obtain the results reported here, and the full R output, are available in an open depository at https://osf.io/jg6eu.
