# Supplementary material for: Predictors of psychological stress and behavioural diversity among captive red panda in Indian zoos and their implications for global captive management
Source: Sci Rep. 2022 Aug 18;12:14034. doi: 10.1038/s41598-022-17872-y (PMC9388642; doi:10.1038/s41598-022-17872-y)
Supplement: Supplementary file 1 — Supplementary Information. [file 41598_2022_17872_MOESM1_ESM.docx]

Supplementary material: Details of individual red pandas sampled for stereotype and behavioural diversity

| Place | Red Panda  ID | Age | Sex | Enclosure  area (m^2^) | # nest | Log/m^2^ | Provision of bamboo |
| --- | --- | --- | --- | --- | --- | --- | --- |
| Zoo 1 | RP1 | 4 | F | 3068 | 2 | 0.002 | High |
| Zoo 1 | RP2 | 6 | F | 192 | 3 | 0.073 | High |
| Zoo 1 | RP3 | 17 | M | 192 | 3 | 0.073 | High |
| Zoo 1 | RP4 | 3 | M | 1560 | 2 | 0.002 | High |
| Zoo 1 | RP5 | 5 | F | 1560 | 2 | 0.002 | High |
| Zoo 1 | RP6 | 5 | M | 1560 | 2 | 0.002 | High |
| Zoo 1 | RP7 | 8 | F | 495 | 3 | 0.042 | High |
| Zoo 1 | RP8 | 3 | F | 2925 | 3 | 0.002 | High |
| Zoo 1 | RP9 | 9 | F | 2925 | 3 | 0.002 | High |
| Zoo 1 | RP10 | 0.6 | M | 2925 | 3 | 0.002 | High |
| Zoo 1 | RP11 | 3 | F | 350 | 1 | 0.017 | High |
| Zoo 1 | RP12 | 11 | M | 322 | 3 | 0.078 | High |
| Zoo 1 | RP13 | 0.8 | F | 322 | 3 | 0.078 | High |
| Zoo 1 | RP14 | 0.8 | M | 322 | 3 | 0.078 | High |
| Zoo 1 | RP15 | 8 | F | 462 | 2 | 0.035 | High |
| Zoo 1 | RP16 | 8 | M | 462 | 2 | 0.035 | High |
| Zoo 1 | RP17 | 3 | F | 400 | 3 | 0.045 | High |
| Zoo 2 | RP18 | 7 | F | 2463 | 1 | 0.016 | Low |
| Zoo 2 | RP19 | 0.9 | M | 2463 | 1 | 0.016 | Low |
| Zoo 2 | RP20 | 0.9 | F | 2463 | 1 | 0.016 | Low |
| Zoo 3 | RP21 | 16 | M | 224 | 4 | 0.134 | Low |
| Zoo 3 | RP22 | 4 | F | 224 | 4 | 0.134 | Low |
| Zoo 3 | RP23 | 3 | M | 448 | 3 | 0.123 | Low |
| Zoo 3 | RP24 | 3 | F | 448 | 3 | 0.123 | Low |
| Zoo 3 | RP25 | 2 | M | 112 | 1 | 0.232 | Low |
| Zoo 3 | RP26 | 2 | F | 112 | 1 | 0.232 | Low |
